# The study of the antinutritional threshold of glycinin in piglets: impact on growth performance, nutrient digestion, allergic reactions, intestinal permeability and morphology

**DOI:** 10.3389/fvets.2025.1646816

**Published:** 2025-08-29

**Authors:** Ning Liu, Xueliang Fu, Bolin Zhang, Qingzhen Zhong, Nan Bao, Zewei Sun

**Affiliations:** ^1^College of Animal Science and Technology, Jilin Agricultural University, Changchun, China; ^2^College of Animal Science and Technology, Qingdao Agricultural University, Qingdao, China

**Keywords:** glycinin, piglets, anti-nutritional threshold, allergic reaction, intestinal permeability

## Abstract

Glycinin is identified as one of the major dietary allergens in soybean. However, the safety threshold of glycinin in piglets have not been clearly understood. The purpose of this study was to investigate the anti-nutritional effects and sensitization threshold of purified glycinin in weaned piglets. In this experiment, 45 21-day-old weaned castrated piglets (Duroc × Landrace × Yorkshire), with a similar initial body weight of 9.95 ± 0.43 kg, were randomly divided into five groups with nine replicates per group. The piglets in the control group were fed a basal diet, while the piglets in the experimental groups were fed diets containing 1, 2, 3 and 4% purified glycinin, respectively, based on the same basal diet. After determination, the glycine content in the diets of each group was 0.704, 8.779, 16.857, 24.934, and 33.011 mg/g, respectively. Samples of feces, serum, digesta, as well as the segments and mucosa of the small intestine, were collected. The measured parameters included the nutrient apparent metabolic rate, digestive enzyme activities, levels of specific immunoglobulin G, histamine (HIS), and D-lactic acid (D-LA) levels, along with small intestinal morphology. The results showed that, compared to the control group, the average daily gain, the average daily feed intake, and the ratio of weight gain to feed intake of the piglets were linearly and quadratically decreased by the inclusion of 2, 3, and 4% glycinin in the diets (*p* < 0.05). In addition, positive skin sensitization, increased erythema diameter, and elevated specific immunoglobulin G titers were induced by glycinin inclusion (*p* < 0.05). In addition, 2, 3, and 4% glycinin administration led to decreases in the apparent nutrient metabolic ratio and digestive enzyme activities (*p* < 0.05). Moreover, the levels of HIS, diamine oxidase, and D-lactic acid in the small intestine were increased in response to 3 and 4% glycinin supplementation (*p* < 0.05). In addition, the reduced D-xylose concentration and damaged intestinal morphology in the piglets observed were induced by the inclusion of 2, 3, and 4% glycinin (*p* < 0.05). The above results indicate that glycinin inclusion at doses above 1% can trigger allergenic effects, including increased erythema diameter, elevated specific immunoglobulin G titers, and higher levels of HIS and D-lactic acid. In contrast, glycinin inclusion at doses above 2% can induce some anti-nutrition effects, such as decreased apparent nutrient metabolic ratio, reduced digestive enzyme activities, and damaged small intestinal morphology. In addition, the information mentioned above implied that the sensitization threshold of glycinin administration in the piglets was 8.779 mg/g and the anti-nutrition threshold was 16.857 mg/g.

## 1 Introduction

Soybean, a high-quality plant protein known for its balanced amino acids and functional properties, is widely used in the food and feed industries (1, 2). To date, various antigenic proteins, including glycinin, *α*-conglycinin, *β*-conglycinin, and *γ*-conglycinin, have been identified in soybean and its products, among which glycinin and β-conglycinin are the main allergenic factors and the most extensively studied (3, 4). Glycinin is the main soybean storage protein, approximately accounting for 40% of the total soybean protein, and consists of six subunits (5, 6). Moreover, glycinin is a heat-insensitive protein whose antigenicity cannot be effectively eliminated or reduced by heat treatment and enzymatic hydrolysis (7). It is well documented that glycinin is one of the major dietary allergens in human and animal diets (8).

It has been demonstrated that allergic reactions in piglets primarily manifest as decreased nutrient digestibility, impaired digestive function, growth retardation, and diarrhea (9). In previous studies, it has been reported that dietary glycinin inclusion could damage small intestinal morphology, destroy immunologic function, and reduce the growth performance of young animals (10, 11). Zhao et al. (12) reported that the average daily weight gain (ADG) and average daily feed intake (ADFI) of piglets were significantly reduced by supplementation with 2.2% purified glycinin. Huang et al. (13) showed that dietary glycinin inclusion at the level of 4% induced diarrhea symptoms, typical skin wheals, and other allergic reactions, as well as anti-nutritional effects, in pigs. However, Sun et al. (2) suggested that dietary supplementation with 2% glycinin did not affect the ADFI of piglets. In addition, in a previous study, it was suggested that 4% purified glycinin administration increased intestinal permeability, accompanied by significant increases in the content of D-lactic acid (D-LA) and diamine oxidase (DAO) in the serum of piglets (14). Moreover, in a previous study, it was shown that 6% purified glycinin administration induced severe diarrhea, decreased growth rate, elevated histamine (HIS) levels, and aggravated intestinal inflammation and barrier dysfunction in piglets (9). The information mentioned above indicates that there may be a dose-dependent sensitization effect in piglets in response to the content of glycinin inclusion in the diet. However, to the best of our knowledge, the anti-nutritional mechanism and safety threshold of soybean antigen proteins in piglets have not been clearly understood.

The objective of the present study was to investigate the effects of different doses of purified glycinin on growth performance, apparent nutrient digestibility, digestive enzyme activities, serum biochemical parameters, gut permeability, and histomorphology in piglets. The results could help determine the threshold value of glycinin inclusion in the diets of piglets, providing scientific guidance for appropriate soybean product incorporation in piglet feed formulations.

## 2 Materials and methods

### 2.1 Animals, diets, and experimental design

A total of 45 21-day-old castrated weaned pigs (Duroc × Landrace × Yorkshire) with a similar initial body weight (9.95 ± 0.43 kg) were equally randomly divided into five groups, including a control group and four experimental groups. The pigs in the control group were fed a basal diet, while the pigs in the experimental groups were provided with diets containing 1, 2, 3 and 4% purified glycinin extracts, respectively. The soybean-extracted glycinin was kindly provided by Professor Shuntang Guo of China Agricultural University. The protein content of glycinin was measured, and its purity was determined to be 86.5% (15). The contents of glycinin in the diets of the five groups were 0.704, 8.779, 16.857, 24.934, and 33.011 mg/g, respectively, which were determined following a previously published method (12). The ingredients and nutrient levels of the diets are shown in Table 1. The diet was formulated to meet the nutrient requirements recommended by the NRC (2012). The experiment was conducted over a 15-day period, during which the piglets had free access to food and water.

**Table 1:** Ingredients and nutrient levels of the experimental diets (%, as-fed basis).

Ingredients	Basal diet	Glycinin group			
		1%	2%	3%	4%
Corn	53.52	54.09	54.63	55.17	55.74
Rice bran meal	3.00	3.00	3.00	3.00	3.00
Cottonseed meal	6.00	5.65	5.30	4.96	4.61
Rapeseed meal	3.00	2.82	2.65	2.48	2.30
Fermented soybean meal	11.00	10.34	9.72	9.09	8.46
Fish meal	4.00	3.76	3.53	3.30	3.07
Meat and bone meal	3.00	2.82	2.65	2.48	2.30
Alfalfa meal	1.00	1.00	1.00	1.00	1.00
Whey powder	5.00	5.00	5.00	5.00	5.00
Glucose	2.00	2.00	2.00	2.00	2.00
Saccharose	3.00	3.00	3.00	3.00	3.00
Soybean oil	2.05	2.05	2.05	2.05	2.05
Mountain flour	0.54	0.54	0.54	0.54	0.54
Sodium chloride	0.65	0.65	0.65	0.65	0.65
Lysine 98%	0.75	0.80	0.80	0.80	0.80
DL-methionine	0.20	0.20	0.20	0.20	0.20
Threonine	0.23	0.23	0.23	0.23	0.23
Tryptophan	0.06	0.05	0.05	0.05	0.05
Mineral and vitamin Premix^1^	1.00	1.00	1.00	1.00	1.00
Glycinin	0.00	1.00	2.00	3.00	4.00
Total	100	100	100	100	100
Nutrition composition					
NE^2^ MJ/kg	10.07	10.11	10.16	10.20	10.24
Lysine, %	1.54	1.57	1.56	1.55	1.55
Methionine, %	0.51	0.51	0.51	0.51	0.51
Cystine, %	0.28	0.28	0.28	0.28	0.28
Methionine+Cystine, %	0.79	0.79	0.79	0.79	0.80
Threonine, %	0.88	0.88	0.88	0.88	0.88
Tryptophan, %	0.25	0.24	0.25	0.25	0.25
Analyzed composition					
Crude protein, %	18.76	18.78	18.79	18.80	18.80
Crude fat, %	4.91	4.88	4.85	4.82	4.79
Ash, %	6.51	6.46	6.42	6.38	6.33
Calcium, %	0.76	0.73	0.71	0.68	0.65
Phosphorus, %	0.62	0.60	0.58	0.56	0.54

^1^Premix provided the following per kg of complete diet for the piglets: VA 28,500 IU, VB_1_ 7.5 mg, VB_2_15 mg, VB_6_ 9 mg, VB_12_ 0.075 mg, VD_3_ 6,000 IU, VE 67.5 IU, VK_3_7.5 mg, niacin 70 mg, folacin 3 mg, pantothenic calcium 37.5 mg, choline 105 mg, biotin 0.375 mg, antioxidants 0.15 mg, Co 1 mg, Cu 145 mg, Fe 155 mg, Mn 75 mg, Zn 125 mg, I 0.3 mg, and Se 0.3 mg. ^2^Values were estimated based on the NRC database (2012) and the determination of the nutrient content of glycinin.

### 2.2 Sample collection

During the 3 days before the end of the experiment, fresh fecal samples from each piglet were separately collected twice per day (8,00 am to 5,00 pm) into sterile bags, then mixed with 10% hydrochloric acid for further analysis of apparent nutrient digestibility. Acid-insoluble ash was used as an intrinsic marker.

On the 15th day of the experiment, the piglets were weighed individually after a 6-h fasting period. After weighing, blood samples were collected from the jugular vein (16). Blood samples from each piglet were collected into 10 mL gel vacuum collection tubes by puncturing the anterior vena cava on days 0, 3, 6, 9, 12, and 15 of the experiment. Subsequently, the blood samples were centrifuged at 2000 rpm/min for 20 min to collect serum and stored at −80°C for immunoglobulin and blood biochemical analysis.

Following the protocols specified in GB/T 39760–2021, all piglets were humanely euthanized on day 15 via intravenous injection of pentobarbital sodium (90 mg/kg) and subsequent jugular exsanguination. After opening the abdomen, the duodenum (extending from the pylorus to the distal end of the pancreatic loop), the jejunum (from the end of the pancreatic loop to Meckel’s diverticulum), and the ileum (from Meckel’s diverticulum to the ileocecal junction) were immediately isolated and rinsed with 5 mL of 0.9% precooled physiological saline. Intestinal segments approximately 3 cm in length were collected and fixed in 4% (w/v) paraformaldehyde for small intestinal histological analysis. Meanwhile, intestinal mucosal samples from the duodenum, jejunum, and ileum were rapidly collected into tubes and stored in liquid nitrogen until analysis.

### 2.3 Growth performance and the incidence of diarrhea

The body weight and feed intake of each piglet were recorded at the beginning and end of the experiment to calculate the ADG, ADFI, and the ratio of body weight gain to feed intake (G: F).

During the experiment, the incidence of diarrhea was defined as the condition in which the water content of fresh feces was greater than or equal to 80%. Then, the incidence of diarrhea was calculated according to the formula reported by Sun et al. (11). The formula was as follows:


$$\begin{array}{l} \text{Diarrhea}\;(\%)=(\begin{array}{l} \text{Number of diarrhea pigs}\times \\ \text{Days of diarrhea} \end{array})/\\ (\begin{array}{l} \text{Total number}\;\\ \text{of pigs}\times \\ \text{Total number}\;\\ \text{of test days} \end{array})\times 100 \end{array}$$

### 2.4 Nutrient apparent metabolic ratio

The dry matter, crude protein, and ether extract of the feed and fecal samples were determined using the methods of the Association of Official Analytical Chemists (AOAC, 2000). For the calculation of the apparent metabolic rate of the dry matter, crude protein, and ether extract in the experimental diets, the following equation was used:

Apparent metabolic rate:


$$(\%)=[1-(A_{1}/A_{2})\times (B_{2}/B_{1})]\times 100$$

where A_1_ is the content of insoluble ash in hydrochloric acid in the feed (%); A_2_ is the content of insoluble ash in hydrochloric acid in the feces (%); B_1_ is the content of the nutrient in the feed (%); B_2_ is the content of the nutrient in the feces (%).

### 2.5 Skin sensitization test

On day 7 of the experiment, 0.5 mg of purified glycinin skin was dissolved in a physiological saline solution, which was intradermally injected into the shaved flank region of the piglets for skin sensitization assessment. Meanwhile, the piglets in the control group were injected with the same amount of physiological saline. The erythema diameter was measured using vernier calipers after 30 min of the intradermal injection. An erythema diameter larger than 5 mm was considered positive for the glycinin-sensitized piglets (17, 18). The criteria for allergic reaction evaluation are shown in Table 2.

**Table 2:** Judgment criteria of piglet allergic reaction.

Rank	Erythema diameter
−	No erythema
±	1–5 mm
+	5–10 mm
++	10–20 mm

### 2.6 The activities of digestive enzymes

The activities of trypsin, lipase, and amylase in the digesta from the duodenum, jejunum, and ileum were measured using commercial kits according to the manufacturers’ instructions. (Nanjing Jian Cheng Bioengineering Research Institute, Nanjing, China).

### 2.7 The concentration of D-xylose

The concentration of D-xylose was determined according to a previous method with minor modifications (19). Briefly, on days 6 and 12 of the trial, D-xylose was orally administered to all piglets at a dose of 0.1 g/kg body weight. One hour after the D-xylose administration, blood samples were collected from the anterior vena cava into heparinized vacuum tubes and centrifuged at 3000 rpm/min for 10 min to obtain serum. The separated serum samples were stored at −20°C and tested with commercial kits (Nanjing Jian Cheng Bioengineering Research Institute, Nanjing, China).

### 2.8 Biochemical parameters in serum and intestinal mucosa

The concentrations of total protein (TP), albumin (ALB), globulin (GLOB), and blood urea nitrogen (BUN) were analyzed using commercial kits (Jiancheng Institute of Biological Technology, Nanjing, China). The level of specific IgG was measured according to the method described by Helm et al. (18). The content of DAO, D-lactic acid (D-LA), and HIS in the mucosa of the duodenum, jejunum, and ileum was determined using commercially available kits according to the manufacturer’s instructions (Shanghai Langdon Biological Technology Co., Ltd., Shanghai, China).

### 2.9 Intestinal morphology

Intestinal morphological measurements were conducted according to the procedures reported by Zhang et al. (20). Briefly, intestinal segments from the duodenum, jejunum, and ileum were collected within 5 min after scarification and rinsed with 0.9% saline solution. Then, these segments were carefully excised, dehydrated through graded concentrations of alcohol and chloroform, embedded in paraffin wax, and cut into cross-sections with a thickness of approximately 5 μm using a microtome. The sections were stained with hematoxylin and eosin for computer-aided light microscope image analysis. Villus height (VH) and crypt depth (CD) were measured using a computer-assisted morphometric system (Bioscan Optimetric, BioSan Inc., Edmonds, WA, United States). A total of 10 well-oriented, intact villus-crypt units were selected in triplicate for each section.

### 2.10 Statistical analysis

All data were checked for a normal distribution and homogeneous variance using Levene’s test. All statistical analyses among the groups were presented as mean ± standard deviation and analyzed using one-way analysis of variance (one-way ANOVA), followed by LSD multiple comparison tests using the SPSS software (IBM SPSS Statistics 20 for Windows). Linear and quadratic regression analyses were conducted to analyze the measured parameters in response to dietary glycinin administration. The relationship among glycinin addition, erythema diameter, the content of IgG, ADG, ADFI, D-xylose concentration, apparent digestibility of crude protein, and apparent digestibility of crude fat was analyzed using Pearson’s correlation analysis. A *p*-value of < 0.05 was considered statistically significant.

## 3 Results

### 3.1 Effect of glycinin on the growth performance and diarrhea incidence of the piglets

As shown in Table 3, compared to the control group (no glycinin addition), the ADFI of the piglets receiving diets containing 1, 2, 3, and 4% glycinin decreased (linear and quadratic, *p* < 0.05). Moreover, the ADG of the piglets fed diets containing 2, 3, and 4% glycinin was significantly lower than that of the control group and the 1% glycinin group (linear and quadratic, *p* < 0.05). In contrast, significant increases in the G: F ratio were observed in response to the inclusion of 2, 3, and 4% glycinin, compared to the control group and the 1% glycinin group (linear and quadratic, *p* < 0.05). The diarrhea rate of the piglets increased from 11.66 to 45.00% when the amount of glycinin was increased from 1 to 3%. The diarrhea rate of the piglets reached 53.33% in response to glycinin administration at a dose of 4%.

**Table 3:** Effect of glycinin administration on the growth performance and diarrhea incidence of the piglets.

Item	Control^1^	Glycinin^1^				SEM^2^	*p*-value		
		1%	2%	3%	4%		Treatment	Linear	Quadratic
ADFI^3^, g/d	554.99 ^a^	537.17^b^	531.75^b^	500.70^c^	489.57^c^	6.005	<0.001	<0.001	<0.001
ADG^3^, g/d	320.13^a^	310.05^a^	230.15^b^	190.08^b^	230.50^b^	12.431	<0.001	<0.001	<0.001
G: F^3^, g/g	0.58^a^	0.58^a^	0.43^b^	0.38^c^	0.47^b^	0.097	<0.001	0.006	0.004
Diarrhea incidence (%)	6.66	11.66	26.66	45.00	53.33	NS	NS	NS	NS

^1^Control group: the piglets were fed the basal diet with no additional glycinin, and the determined dose of glycinin in the diet was 0.704 mg/g. 1% glycinin group: the determined dose of glycinin in the diet was 8.779 mg/g. 2% glycinin group: the determined dose of glycinin in the diet was 16.857 mg/g. 3% glycinin group: the determined dose of glycinin in the diet was 24.934 mg/g. 4% glycinin group: the determined dose of glycinin in the diet was 33.011 mg/g. ^2^SEM, standard error of the mean. Data are expressed as means and SEM. ^3^ADFI, average daily feed intake; ADG, average daily gain; G: F, the ratio of feed consumption to body weight gain. ^a–c^Data with different lowercase letters in the same line indicate significant differences (*p* < 0.05).

### 3.2 Effect of glycinin on skin sensitization in the piglets

As presented in Table 4, the piglets fed diets containing 1, 2, 3, and 4% glycinin exhibited obvious positive skin sensitization reactions. The erythema diameters of the piglets in the 1, 2, 3, and 4% glycinin groups were significantly larger than those in the control group (linear and quadratic, *p* < 0.05).

**Table 4:** Effect of glycinin administration on skin sensitization and erythema diameter in the piglets.

Item	Saline^1^	Glycinin group^1^				SEM^2^	*p*-value		
		1%	2%	3%	4%		Treatment	Linear	Quadratic
Rank*	−	+	++	++	++	NS	NS	NS	NS
Number of positive reactions	0/9	8/9	8/9	9/9	8/9	NS	NS	NS	NS
Erythema diameter, mm	4.05^c^	11.05^b^	12.02^b^	12.3^b^	14.14^a^	0.828	<0.001	<0.001	<0.001

^1^Control group: the piglets were fed a basal diet and injected with saline as a negative control. 1% glycinin group: the determined dose of glycinin in the diet was 8.779 mg/g, and the piglets were injected with 0.5 mg purified glycinin dissolved in 0.5 mL physical saline. 2% glycinin group: the determined dose of glycinin in the diet was 16.857 mg/g, and the piglets were injected with 0.5 mg purified glycinin dissolved in 0.5 mL physical saline. 3% glycinin group: the determined dose of glycinin in the diet was 24.934 mg/g, and the piglets were injected with 0.5 mg purified glycinin dissolved in 0.5 mL physical saline. 4% glycinin group: the determined dose of glycinin in the diet was 33.011 mg/g, and the piglets were injected with 0.5 mg purified glycinin dissolved in 0.5 mL physical saline. ^2^SEM, standard error of the mean. Data are expressed as means and SEM. ^a–c^Data with different lowercase letters in the same line indicate significant differences (*p* < 0.05).

### 3.3 Effect of glycinin on the concentration of specific IgG antibodies

As presented in Table 5, on days 9 and 15 of the experiment, the piglets sensitized with 2, 3, and 4% glycinin exhibited significant increases in glycinin-specific IgG levels compared to the control group and the 1% glycinin group (linear and quadratic, *p* < 0.05). Moreover, the glycinin-specific IgG levels of the piglets fed diets containing 3 and 4% glycinin were significantly higher compared to the control, 1, and 2% glycinin groups on days 3, 6, and 12 (linear and quadratic, *p* < 0.05).

**Table 5:** Specific IgG against glycinin in the piglets.

Item	Control^1^	Glycinin group^1^				SEM^2^	*p*-value		
		1%	2%	3%	4%		Treatment	Linear	Quadratic
IgG^3^, OD_495_									
Day 3	0.46^b^	0.51^ab^	0.50^ab^	0.57^a^	0.58^a^	0.017	0.126	0.008	0.032
Day 6	0.48^c^	0.53^bc^	0.53^bc^	0.61^b^	0.71^a^	0.021	0.001	<0.001	<0.001
Day 9	0.50^c^	0.53^bc^	0.56^b^	0.72^a^	0.77^a^	0.025	<0.001	<0.001	<0.001
Day 12	0.53^c^	0.56^c^	0.59^bc^	0.77^ab^	0.81^a^	0.037	0.018	0.001	0.003
Day 15	0.55^c^	0.58^bc^	0.63^b^	0.81^a^	0.87^a^	0.031	<0.001	<0.001	<0.001

^1^Control group: the piglets were fed a basal diet with no additional glycinin, and the determined dose of glycinin in the diet was 0.704 mg/g. 1% glycinin group: the determined dose of glycinin in the diet was 8.779 mg/g. 2% glycinin group: the determined dose of glycinin in the diet was 16.857 mg/g. 3% glycinin group: the determined dose of glycinin in the diet was 24.934 mg/g. 4% glycinin group: the determined dose of glycinin in the diet was 33.011 mg/g. ^2^SEM, standard error of the mean. Data are expressed as means and SEM. ^3^IgG = immunoglobulin G. ^a–c^Data with different lowercase letters in the same line indicate significant differences (*p* < 0.05).

### 3.4 Effect of glycinin on blood biochemical indexes

As depicted in Table 6, reductions in TP and an increase in BUN were observed in the serum of the weaned piglets fed diets containing 2, 3, and 4% glycinin compared to the control group (linear and quadratic, *p* < 0.05). In addition, 4% glycinin administration led to a decreased concentration of ALB in the serum of the piglets (*p* < 0.05). However, no differences in GLOB were observed between the control and glycinin-administration groups (*p* > 0.05).

**Table 6:** Effect of glycinin addition on the serum biochemical indexes of the piglets.

Item	Control^1^	Glycinin group^1^				SEM^2^	*p*-value		
		1%	2%	3%	4%		Treatment	Linear	Quadratic
TP^3^, g/L	67.03^a^	65.08^ab^	60.4^b^	57.08^c^	55.94^c^	1.117	<0.001	<0.001	<0.001
ALB^3^, g/L	41.65^a^	41.03^a^	38.71^ab^	38.80^ab^	31.40^b^	1.308	0.077	0.010	0.019
GLOB^3^, g/L	25.37	24.05	21.69	18.28	24.53	1.261	0.419	0.419	0.304
BUN^3^, mmol/L	7.98^d^	8.61^d^	9.55^c^	11.65^b^	12.69^a^	0.423	<0.001	<0.001	<0.001

^1^Control: the piglets were fed a basal diet with no additional glycinin, and the determined dose of glycinin in the diet was 0.704 mg/g. 1% glycinin group: the determined dose of glycinin in the diet was 8.779 mg/g. 2% glycinin group: the determined dose of glycinin in the diet was 16.857 mg/g. 3% glycinin group: the determined dose of glycinin in the diet was 24.934 mg/g. 4% glycinin group: the determined dose of glycinin in the diet was 33.011 mg/g. ^2^SEM, standard error of the mean. Data are expressed as means and SEM. ^3^TP = total protein; ALB = albumin; GLOB = globulin; BUN = urea nitrogen. ^a–d^Data with different lowercase letters in the same line indicate significant differences (p < 0.05).

### 3.5 Effect of glycinin on the metabolic rate of nutrients

There were no significant differences in the metabolic rate of dry matter, crude protein, and ether extract in the diets between the control group and the 1% glycinin group (*p* > 0.05; Table 7). However, the metabolic rate of dry matter decreased with glycinin inclusion at levels of 2, 3, and 4% (linear and quadratic, *p* < 0.05). In addition, the piglets fed diets containing 3 and 4% glycinin showed a significant decrease in the metabolic rate of dry matter and crude protein compared to those in the control, 1, and 2% glycinin groups (linear and quadratic, *p* < 0.05).

**Table 7:** Effect of glycinin addition on the nutrient apparent metabolic ratio in the piglets (%).

Item	Control^1^	Glycinin group^1^				SEM^2^	*p*-value		
		1%	2%	3%	4%		Treatment	Linear	Quadratic
Dry matter	81.35^a^	80.48^ab^	78.14^b^	75.65^c^	72.56^d^	0.906	<0.001	<0.001	<0.001
Crude protein	81.21^a^	80.60^ab^	78.36^b^	75.51^c^	71.68^d^	0.993	<0.001	<0.001	<0.001
Ether extract	79.83^a^	78.25^ab^	76.56^bc^	75.12^c^	73.12^d^	0.662	<0.001	<0.001	<0.001

^1^Control: the piglets were fed a basal diet with no additional glycinin, and the determined dose of glycinin in the diet was 0.704 mg/g. 1% glycinin group: the determined dose of glycinin in the diet was 8.779 mg/g. 2% glycinin group: the determined dose of glycinin in the diet was 16.857 mg/g. 3% glycinin group: the determined dose of glycinin in the diet was 24.934 mg/g. 4% glycinin group: the determined dose of glycinin in the diet was 33.011 mg/g. ^2^SEM, standard error of the mean. Data are expressed as means and SEM. ^a–d^Data with different lowercase letters in the same line indicate significant differences (*p* < 0.05).

### 3.6 Effects of glycinin on digestive enzyme activity in the small intestine

**Figure 1:**
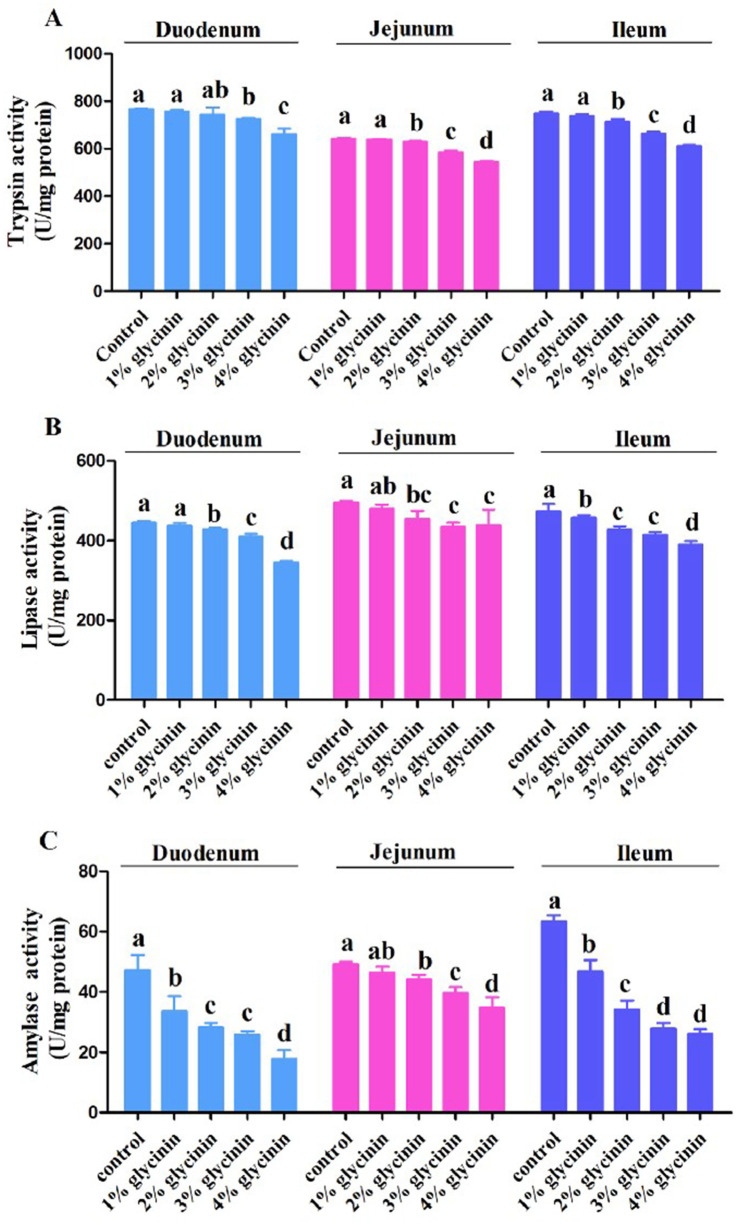
Effect of glycinin administration on trypsin (**A**), lipase (**B**) and amylase (**C**) activities in the duodenum, jejunum, and ileum of the piglets. In the control group, the piglets were fed a basal diet with no additional glycinin, and the determined dose of glycinin in the diet was 0.704 mg/g. In the 1% glycinin group, the determined dose of glycinin in the diet was 8.779 mg/g; in the 2% glycinin group, the determined dose of glycinin in the diet was 16.857 mg/g; in the 3% glycinin group, the determined dose of glycinin in the diet was 24.934 mg/g; and in the 4% glycinin group, the determined dose of glycinin in the diet was 33.011 mg/g. SEM, standard error of the mean. Data are expressed as means and SEM. Data with different lowercase letters in the same line indicate significant differences (*p* < 0.05).

As shown in Figure 1, 1% glycinin administration decreased the activity of amylase in the duodenum and the activities of lipase and amylase in the ileum compared to the control group (*p* > 0.05). Compared to the control group, the activities of trypsin, lipase, and amylase in the duodenum, jejunum, and ileum were significantly reduced in response to glycinin inclusion at levels of 2, 3, and 4% (*p*<0.05), except for trypsin activity in the duodenum (*p* > 0.05).

### 3.7 Effects of glycinin on DAO and D-LA contents in the small intestine

**Figure 2:**
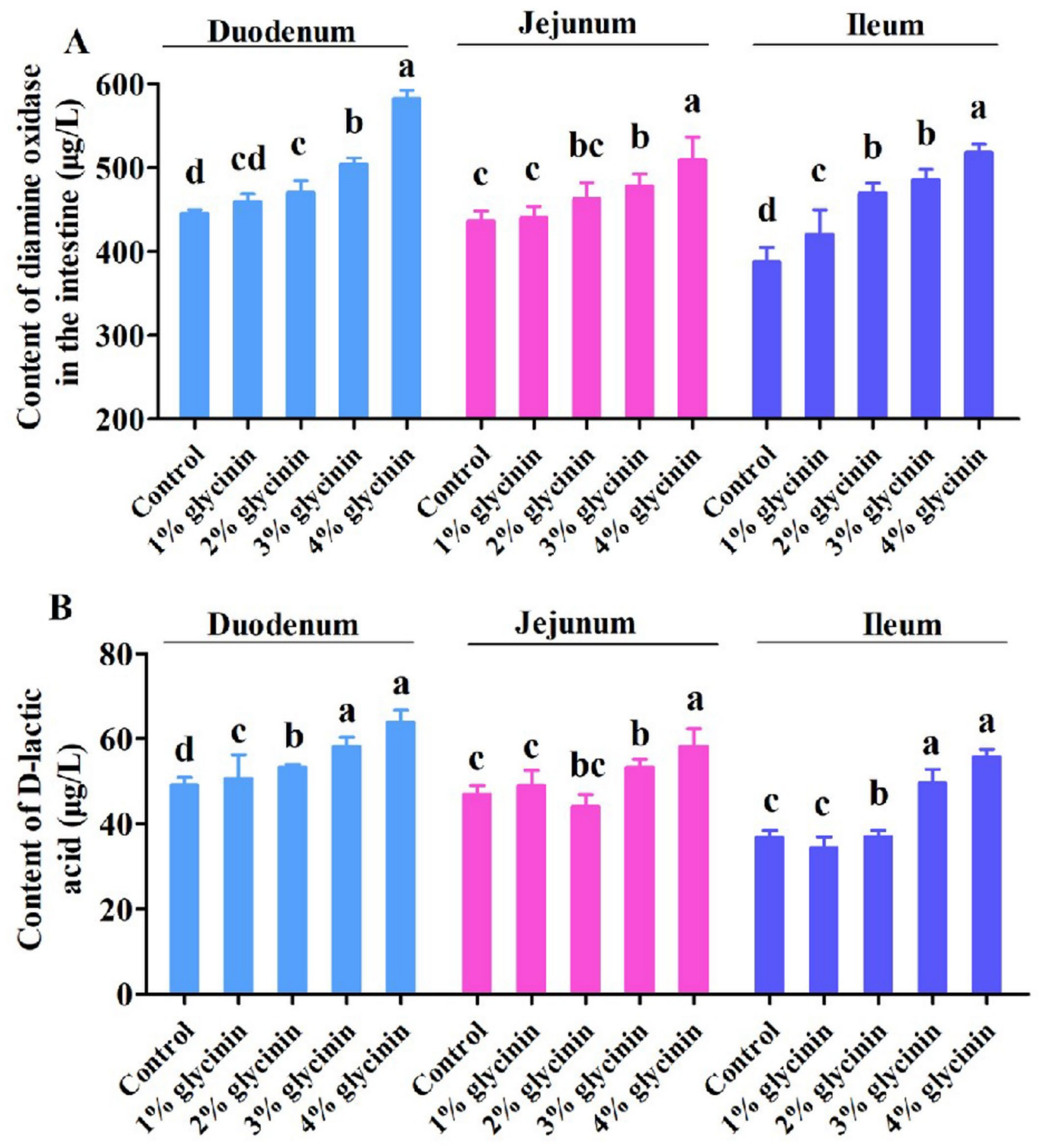
Effect of glycinin administration on the contents of diamine oxidase (**A**) and D-lactic acid (**B**) in the small intestinal mucosa of the piglets. In the control group, the piglets were fed a basal diet with no additional glycinin, and the determined dose of glycinin in the diet was 0.704 mg/g. In the 1% glycinin group, the determined dose of glycinin in the diet was 8.779 mg/g; in the 2% glycinin group, the determined dose of glycinin in the diet was 16.857 mg/g; in the 3% glycinin group, the determined dose of glycinin in the diet was 24.934 mg/g; and in the 4% glycinin group, the determined dose of glycinin in the diet was 33.011 mg/g. SEM, standard error of the mean. Data are expressed as means and SEM. Data with different lowercase letters in the same line indicate significant differences (*p* < 0.05).

The concentrations of DAO and D-LA in the small intestine of the piglets are shown in Figure 2. The results showed that the increased levels of DAO in the ileum and D-LA in the duodenum were induced by 1% glycinin administration compared to the control group (*p*<0.05). Moreover, dietary supplementation with 2, 3, and 4% glycinin significantly increased the content of DAO and D-LA in the duodenum, jejunum, and ileum of the piglets compared to the control group (*p* < 0.05).

### 3.8 Effect of glycinin on the absorption capacity of D-xylose

**Figure 3:**
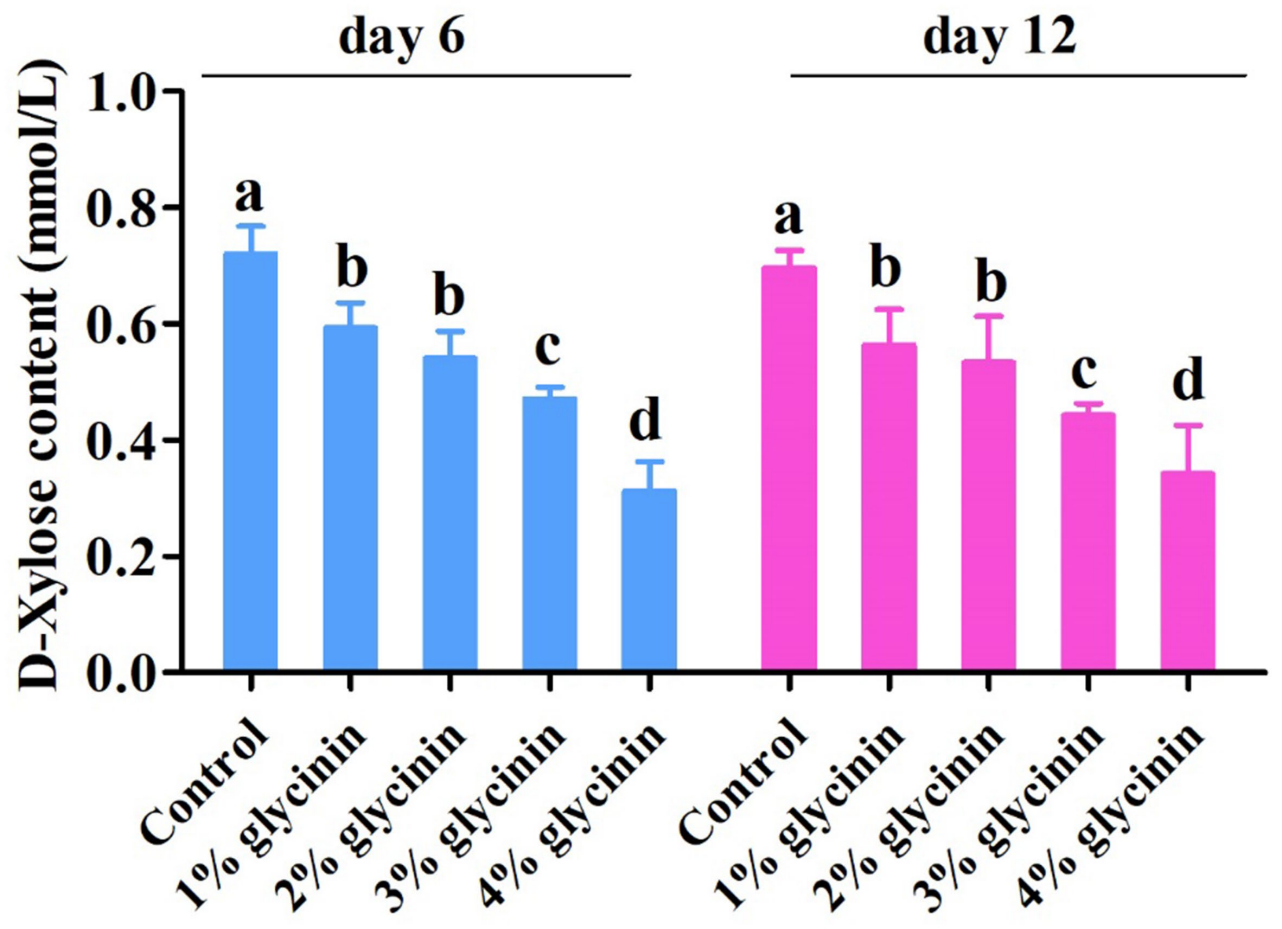
Effect of glycinin administration on the contents of D-xylose in the serum of the piglets. In the control group, the piglets were fed a basal diet with no additional glycinin, and the determined dose of glycinin in the diet was 0.704 mg/g. In the 1% glycinin group, the determined dose of glycinin in the diet was 8.779 mg/g; in the 2% glycinin group, the determined dose of glycinin in the diet was 16.857 mg/g; in the 3% glycinin group, the determined dose of glycinin in the diet was 24.934 mg/g; and in the 4% glycinin group, the determined dose of glycinin in the diet was 33.011 mg/g. SEM, standard error of the mean. Data are expressed as means and SEM. Data with different lowercase letters in the same line indicate significant differences (*p* < 0.05).

The concentration of D-xylose in the serum of the piglets on days 6 and 12 of the trial is presented in Figure 3. The concentration of D-xylose in the serum was lower in the piglets fed 3 and 4% glycinin-supplemented diets compared to those in the control,1, and 2% glycinin groups (*p* < 0.05). In addition, the dietary inclusion of 1 and 2% glycinin also linearly and quadratically reduced D-xylose levels compared to the control group (*p* < 0.05). However, there were no differences in D-xylose levels in the serum between the 1 and 2% glycinin groups (*p* < 0.05). Moreover, the concentration of serum D-xylose in the 4% glycinin group was lower than that in the 3% glycinin administration group (*p*<0.05).

### 3.9 Effect of glycinin on HIS content in the small intestine

**Figure 4:**
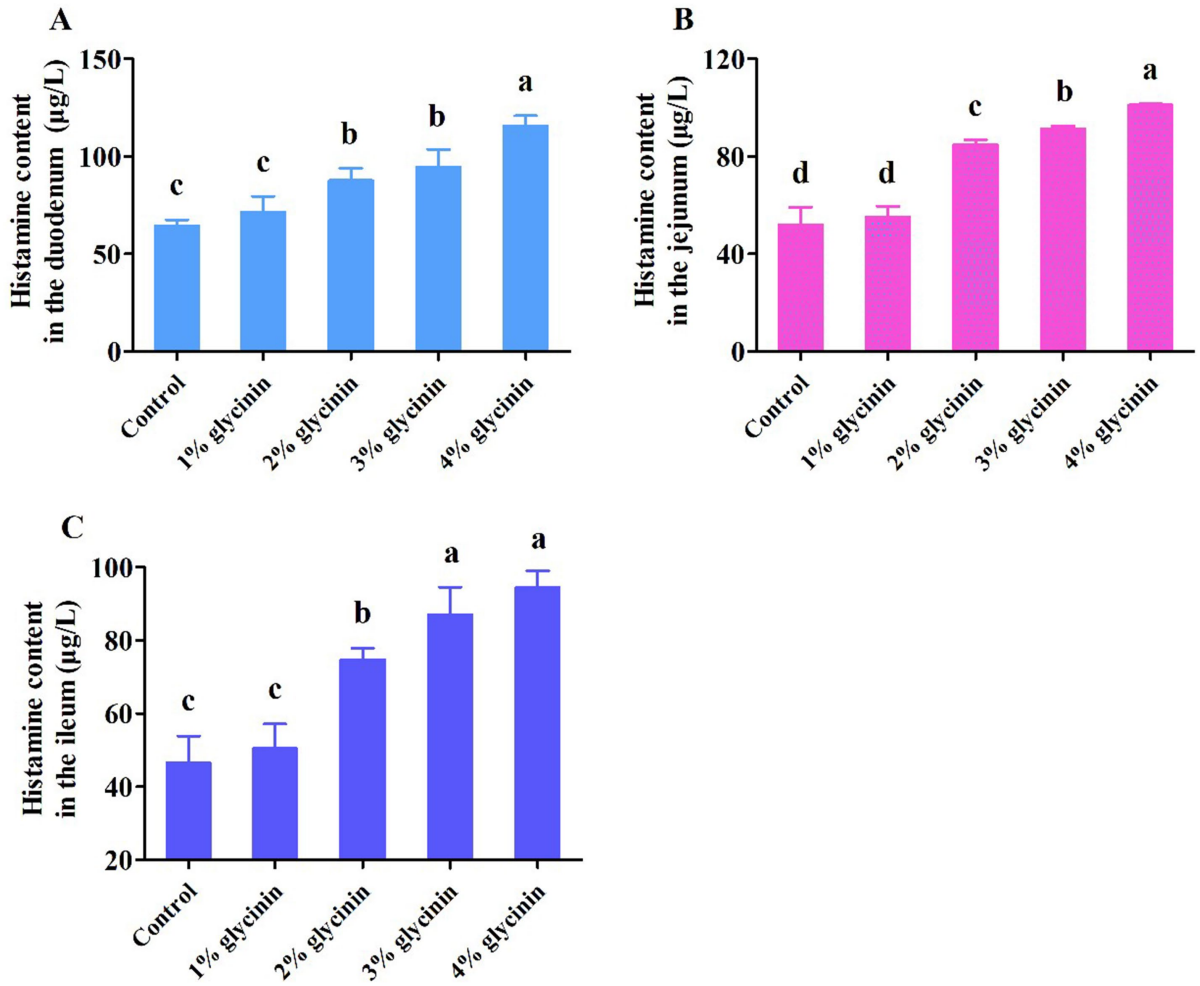
Effect of glycinin administration on histamine content in the mucosa of the duodenum **(A)**, jejunum **(B)**, and ileum **(C)** of the piglets. In the control group, the piglets were fed a basal diet with no additional glycinin, and the determined dose of glycinin in the diet was 0.704 mg/g. In the 1% glycinin group, the determined dose of glycinin in the diet was 8.779 mg/g; in the 2% glycinin group, the determined dose of glycinin in the diet was 16.857 mg/g; in the 3% glycinin group, the determined dose of glycinin in the diet was 24.934 mg/g; and in the 4% glycinin group, the determined dose of glycinin in the diet was 33.011 mg/g. SEM, standard error of the mean. Data are expressed as means and SEM. Data with different lowercase letters in the same line indicate significant differences (*p* < 0.05).

As presented in Figure 4, compared to the control group and 1% glycinin supplementation, the addition of glycinin at levels of 2, 3, and 4% resulted in higher HIS content in the duodenum, jejunum, and ileum (*p* < 0.05). However, no differences in HIS content in the duodenum, jejunum, and ileum were observed between the control group and the 1% glycinin administration group (*p* > 0.05). In addition, the dietary inclusion of 3 and 4% glycinin significantly increased HIS content compared to the 2% glycinin group (*p* < 0.05).

### 3.10 Effect of glycinin on the intestinal morphology of the small intestine

**Figure 5:**
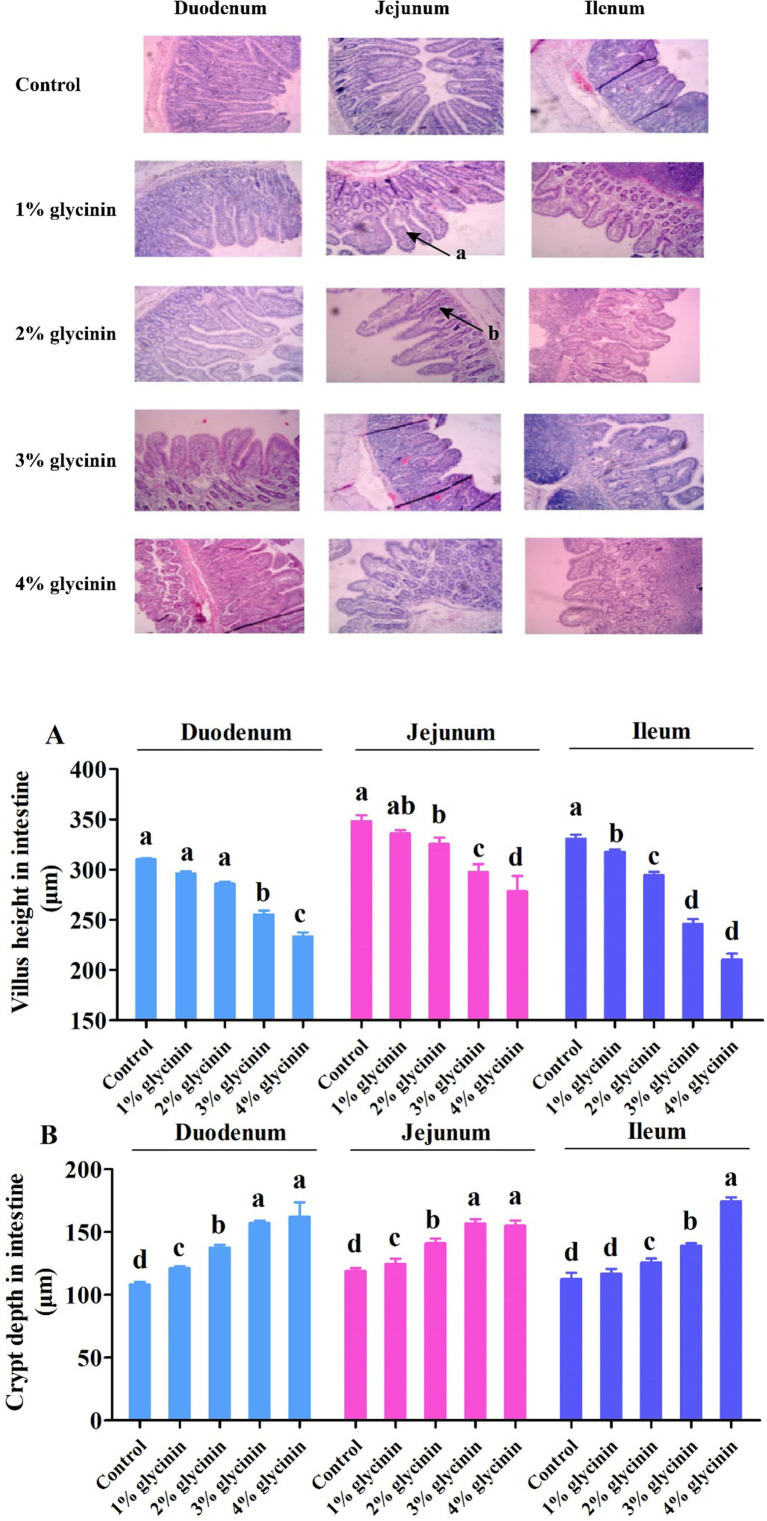
Effect of glycinin administration on villus height **(A)** and crypt depth **(B)** in the small intestine of the piglets. (a) The damaged intestinal villus; (b) the deeper intestinal crypt. In the control group, the piglets were fed a basal diet with no additional glycinin, and the determined dose of glycinin in the diet was 0.704 mg/g. In the 1% glycinin group, the determined dose of glycinin in the diet was 8.779 mg/g; in the 2% glycinin group, the determined dose of glycinin in the diet was 16.857 mg/g; in the 3% glycinin group, the determined dose of glycinin in the diet was 24.934 mg/g; and in the 4% glycinin group, the determined dose of glycinin in the diet was 33.011 mg/g. SEM, standard error of the mean. Data are expressed as means and SEM. Data with different lowercase letters in the same line indicate significant differences (*p* < 0.05).

The results showed that, compared to the control group, the shorter VH observed in the duodenum, jejunum, and ileum, as well as the deeper CD in the duodenum and jejunum, was induced in response to 1, 2, 3, and 4% glycinin supplementation (Figure 5, *p*<0.05). In addition, the ileal CD of the piglets fed diets containing 2, 3, and 4% glycinin was greater than that of the control group and the 1% glycinin group (*p*<0.05). Moreover, the piglets fed a 3% glycinin diet exhibited a significant decrease in VH and increases in CD in the duodenum, jejunum, and ileum compared to those fed a 2% glycinin diet (*p*<0.05).

### 3.11 Correlation between glycinin administration and sensitization reaction, as well as anti-nutritional parameters

**Figure 6:**
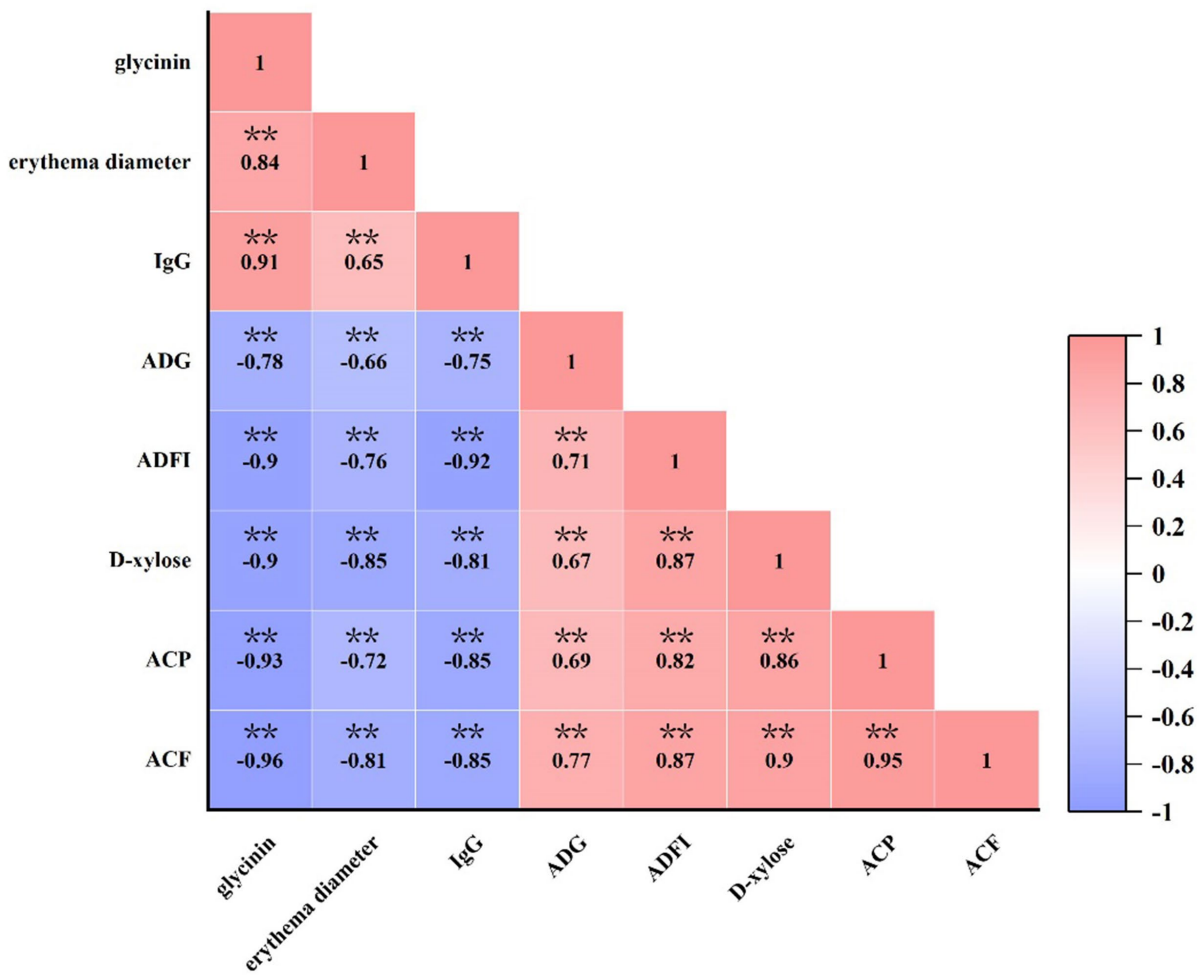
Correlation between glycinin administration and growth performance, IgG and D-xylose concentrations, and the apparent metabolite ratio of crude protein and crude fat in the piglets. Heatmaps depict Pearson’s correlation coefficients. Deep red and dark blue indicate stronger correlations with significant *p*-values, while light red and light blue represent weaker correlations and p-values. IgG, immunoglobulin G; ADFI, average daily feed intake; ADG, average daily gain; ACP, apparent metabolite ratio of crude protein; ACF, apparent metabolite ratio of crude fat. **p < 0.01.

As presented in Figure 6, there was a significant positive correlation between the dose of glycinin administration and the erythema diameter, as well as the concentration of IgG (*p* < 0.05). In contrast, a negative correlation was observed between the dose of glycinin administration and growth performance (ADG and ADFI), the content of D-xylose, and apparent metabolite rates (CP and CF) (*p* < 0.05). Moreover, it was observed that the parameters of sensitization, including erythema diameter and IgG content, were also negatively correlated with growth performance and nutrient apparent metabolite rates (*p* < 0.05).

## 4 Discussion

Soybeans, rich in protein and balanced amino acids, are widely consumed as a high-quality plant protein raw material by both humans and animals (9, 21). Soybean allergy, mediated by several heat-stable anti-nutritional factors, is a serious health threat to sensitive individuals, potentially causing allergic diarrhea and damaging intestinal morphology and the intestinal mucosal barrier (22). Unfortunately, glycinin accounts for approximately 30% of the total soybean protein and has been identified as one of the major allergens in soybeans (23). In a previous study, it was demonstrated that 4 and 8% glycinin (purity > 85%) could induce a lower ADG in weaned piglets (2). Similarly, it was also reported that body weight and average daily gain were decreased, and diarrhea incidence was increased when piglets were fed a diet containing 4% purified glycinin or *β*-conglycinin (purity 80–85%) (13). Consistent with these previous studies, our results revealed that the ADG and G: F ratio of the piglets in the 2, 3, and 4% glycinin groups were significantly lower than those in the control group and the 1% glycinin group. Moreover, significant differences in ADFI were observed in the piglets sensitized with 1, 2, 3, and 4% glycinin administration compared to those in the control group. However, 1% glycinin inclusion did not affect the ADG and G: F ratio of the piglets compared to the control group. Diarrhea incidence rose progressively from 11.66 to 45.00% as the dietary glycinin level increased from 1 to 3%. In our present study, the piglets fed diets containing 1, 2, 3, and 4% glycinin showed increased skin sensitization. In addition, a previous study reported that administration of purified glycinin at (90.6% purity) 2,500 μg/kg BW reduced the ADG, ADFI, and G: F ratio of piglets compared to the control group (24). In line with this, Zhao et al. (12) also found that dietary supplementation with 2.2% glycinin (purity >95%) impaired growth performance and increased diarrhea incidence in piglets. Moreover, a previous study showed that administration of purified glycinin induced the occurrence of diarrhea and typical cutaneous skin flare responses in piglets compared to the control group with no glycinin treatment (13). The skin prick test evaluates mast cell activation and subsequent biological responses induced by allergens (25), establishing it as the gold-standard diagnostic tool for food allergies (26). In our study, the results of the skin test were positive in the glycinin-sensitized pigs, confirming the allergenicity of soybean-derived glycinin. The allergen-sensitizing properties assessed by skin pick tests have also been reported by Huang et al. (13) and Helm et al. (18). Taken together, based on the results of our study, the information mentioned above indicates that inclusion of glycinin above 1% could induce hypersensitivity reactions in piglets.

It has been revealed that piglets are particularly susceptible to the anti-nutritional factors in soybeans, which may impair gut function and trigger diarrhea after weaning. In addition, dietary inclusion of glycinin induced a significant decrease in the apparent digestibility of dry matter and protein (27). However, the digestive and absorptive capacity of the intestine is highly dependent on the activities of digestive enzymes (28). Consistent with this, our results demonstrated that 1% glycinin inclusion in diets decreased the activity of amylase in the duodenum and ileum. The activities of trypsin, lipase, and amylase in the duodenum, jejunum, and ileum were significantly reduced by glycinin inclusion at levels of 2, 3, and 4% compared to the control group. Accordingly, compared to the control group, the dietary inclusion of glycinin at levels of 2, 3, and 4% led to decreases in the apparent digestibility of dry matter, crude protein, and crude lipid (ether extract). The results mentioned above indicate that glycinin inclusion above 1% could decrease apparent nutrient digestibility, which may be associated with reduced enzyme activities.

Serum levels of immunoglobulin (IgG, IgA, and IgM) are key indicators of humoral immune function (29–31). It has been reported that glycinin absorbed into the bloodstream through small intestinal epithelial cells induces the production of large quantities of IgG antibodies in the supernatant fluid of blood (18, 32). It was reported that significantly elevated levels of IgG in crossbred piglets were triggered in response to 4 and 8% glycinin (purity>85%) sensitization (11). Following antigen challenge, serum IgG levels were significantly elevated in piglets fed 2,500 μg glycinin per kg BW (purity 90.6%) (24). In agreement with this, in our present study, the piglets sensitized with 2, 3, and 4% glycinin exhibited a significant increase in glycinin-specific IgG levels compared to the control group and the 1% glycinin group. This suggests that diets containing more than 2% glycinin can cause allergic reactions and serious sensitization reactions.

DAO is a relatively stable marker enzyme, and its content is closely correlated with changes in villus height, nucleic acid, and protein synthesis in small intestinal mucosal cells (33, 34). D-LA, an index of small intestine wall permeability, is a byproduct of bacterial fermentation in the small intestine. The content of D-LA can reflect changes in the intestinal barrier function (35). In previous studies, it was demonstrated that the concentrations of DAO and D-LA were significantly increased by dietary supplementation with 4% or 6% glycinin (purity 90.6%) compared to the control group (14, 36). Consistent with this, it was observed that the addition of glycinin in the diets of the piglets significantly increased the content of DAO and D-LA in the duodenum, jejunum, and ileum. Compared to the 1% glycinin group, the content of DAO in the ileum and D-LA in the duodenum was significantly increased. In addition, the results of a previous study revealed that the contents of DAO and D-LA in glycinin-fed piglets were increased, which is consistent with our results regarding the glycinin-induced impairment of small intestinal permeability and barrier function in piglets (9, 37). Gastrointestinal food allergy represents an IgE-mediated hypersensitivity reaction triggered by dietary antigens (38). Elevated HIS release from the gastrointestinal mucosa following food allergen sensitization serves as a reliable diagnostic marker for food allergy (38). In general, sensitized animals exhibited increased HIS release from the intestinal mucosa (39). After a 4% glycinin (purity 80–85%) challenge, increased HIS content was observed in the duodenum, jejunum, and ileum of glycinin-sensitized piglets compared to the control group (13). Wu et al. (24) reported that pigs ingesting 500 or 2, 500 μg of glycinin per kg body weight (purity 90.6%) exhibited higher HIS levels in the duodenum and ileum. Similarly, in our present study, compared to the control group and 1% glycinin group, the addition of glycinin at levels of 2, 3, and 4% resulted in higher HIS content in the duodenum, jejunum, and ileum. Moreover, in a previous study, it was reported that administration of 4% or 8% glycinin induced higher concentrations of HIS in the jejunum and ileum of pigs compared to the control group (11).

The intestine, a structural basis for the immune barrier, can protect the body from damage and absorb nutrients for animals. The morphology of the intestinal mucosa, particularly the structure of villi and crypts, determines the digestive function and nutrient absorption efficiency of the small intestine (40). Accumulated evidence has demonstrated that high levels of glycinin decrease VH and increase CD in the duodenum, jejunum, and ileum of piglets, mice, and turbot (27, 41, 42). In the present study, the piglets in the glycinin**-**supplemented groups had shorter VH and deeper CD than those in the control group without glycinin treatment. Briefly, compared to the control group, VH in the duodenum, jejunum, and ileum significantly increased, while CD in the duodenum and jejunum significantly decreased in the glycinin-supplemented groups. In addition, Wu et al. (24) reported that piglets exhibited intestinal villus changes after an oral challenge with 500 or 2,500 μg of glycinin per kg body weight (purity 90.6%). A similar observation was reported by Miller et al. (43), whose results found that these antigenic proteins disrupted gastrointestinal morphology, as evidenced by reduced VH, increased CD, and impaired nutrient digestion and absorption compared to the control group. Moreover, it has been demonstrated that transient hypersensitivity to soybean antigens induces villus atrophy and crypt hypertrophy (44), indicating that glycinin administration could lead to damage to intestinal morphology.

## 5 Conclusion

Glycinin inclusion at doses above 8.779 mg/g induced the occurrence of skin sensitization, as evidenced by larger erythema diameter and elevated levels of glycinin-specific IgG. In contrast, glycinin inclusion at doses above 16.857 mg/g impaired growth performance, reduced nutrient digestion, induced allergic sensitization, and damaged the intestinal permeability and morphology of the piglets, implying that the sensitization threshold of glycinin administration in the piglets was 8.779 mg/g and the anti-nutrition threshold was 16.857 mg/g.

## Data Availability

The datasets presented in this study can be found in online repositories. The names of the repository/repositories and accession number(s) can be found in the article/supplementary material.
